# Overexpression of SLC6A1 associates with drug resistance and poor prognosis in prostate cancer

**DOI:** 10.1186/s12885-020-06776-7

**Published:** 2020-04-06

**Authors:** Chaojiang Chen, Zhiduan Cai, Yangjia Zhuo, Ming Xi, Zhuoyuan Lin, Funeng Jiang, Zezhen Liu, Yueping Wan, Yu Zheng, Jianxin Li, Xing Zhou, Jianguo Zhu, Weide Zhong

**Affiliations:** 1 Guangdong Provincial Institute of Nephrology, Nanfang Hospital, Southern Medical University, Guangzhou, 510515 China; 2 Department of Urology, Guangdong Key Laboratory of Clinical Molecular Medicine and Diagnostics, Guangzhou First People’s Hospital, School of Medicine, South China University of Technology, Guangzhou, 510180 China; 3 Department of Urology, The Second Affiliated Hospital of Guangzhou Medical University, Guangzhou Medical University, Guangzhou, China; 4 Department of Urology, Huadu District People’s Hospital, Southern Medical University, Guangzhou, 510800 China; 5 Department of Urology, Guizhou Provincial People’s Hospital, The Affiliated Hospital of Guizhou Medical University, Guiyang, 550002 China; 6 Urology Key Laboratory of Guangdong Province, The First Affiliated Hospital of Guangzhou Medical University, Guangzhou Medical University, Guangzhou, 510230 China

**Keywords:** Prostate cancer, Solute carrier family 6 member 1, Prognosis, Chemotherapy resistance

## Abstract

**Background:**

Solute Carrier Family 6 Member 1 (SLC6A1) has been identified as a cancer-promoting gene in various human cancers, such as clear cell renal cell carcinoma and ovarian cancer. However, its roles in prostate cancer (PCa) has not been fully elucidated. The aim of this study was to investigate the expression and clinical significance of SLC6A1 in PCa tissues and its effect on drug resistance to docetaxel in PCa.

**Methods:**

Expression patterns of SLC6A1 protein in PCa tissues were examined by immunohistochemistry based on Tissue microarray. Associations of SLC6A1 protein expression with various clinicopathological features and patients’ prognosis of PCa were also statistically evaluated based on TCGA data. Roles of SLC6A1 deregulation in prostate carcinogenesis and drug resistance was further determined in vitro and in vivo experiments.

**Results:**

Based on TCGA Dataset, SLC6A1 expression was markedly higher in patients with high Gleason score, advanced clinical stage and positive biochemical recurrence than those with control features (all *P* < 0.05). Both unvariate and multivariate analyses demonstrated that SLC6A1 expression was significantly associated with biochemical recurrence-free survival in PCa patients. In addition, enforced expression of SLC6A1 effectively promoted cell proliferation, migration and invasion of PCa cells in vitro. Moreover, the inhibition of SLC6A1 suppressed the tumor growth in vivo. Additionally, immunohistochemical notches of PCNA and MMP-9 in the low-expression cluster were pointedly lower compared to those of NC group. Finally, the cell viability revealed that the overexpression of SLC6A1 obviously promoted the PCa cell resistant to docetaxel (DTX), and the transplanted tumor in the overexpression group had no significant reduction compared with the untreated group.

**Conclusions:**

Our data suggest that SLC6A1 overexpression may be associated with aggressive progression and short biochemical recurrence-free survival of PCa, and may be related to the resistance to docetaxel therapy.

## Background

Prostate cancer, the fourth common malignancy in Europe [1], is the largest annual increase (26%) of men and the second (9%) in deaths in the United States [2]. Because of PSA screening and MRI examination, prostate cancer can be diagnosed and treated at earliest [3]. Despite of the effective treatments, including castration, radical prostatectomy, and high-dose radiotherapy, 20–30% of patients with prostate cancer may recur biochemically within 5–10 years and progress to hormone-resistant prostate cancer (CRPC) [4–6].

Docetaxel is the first-line medication permitted by FDA for the treatment of CRPC [7]. However, clinical evidence show that only 48% of patients with prostate cancer may respond to the treatment of docetaxel plus prednisone, with an average survival improvement of two and half months over the control group [8]. Therefore, it is of great clinical significance to identify molecular biomarkers which can predict the patients’ response to the treatment of docetaxel, in order to assist in screening the responsive prostate cancer patients to docetaxel and thus potentially benefit individualized therapy of prostate cancer in a daily clinical setting.

Ion channel and transporter (ICT), a new type of membrane proteins, has been observed to be expressed in various human cancers, such as prostate cancer, which expressed KCNA3(Potassium voltage-gated channel, Shaker-related subfamily, member 3) [9] and TRPV6(Transient receptor potential cation channel, subfamily V, member 6) [10] in primary tumors. ICT may play a role in regulating the malignancy of cancer cells, including cell proliferation, migration, invasiveness and apoptosis. As a member of ICT,SLC6A1 encodes sodium- and chloride-dependent gamma amino-butyric acid (GABA) carrier (GAT-1), and restores it to presynaptic terminals by eliminating GABA with synaptic cleft [11]. SLC6A1 has been indicated to be involved into several diseases of the nervous system [12], such as epilepsy [13], schizophrenia [14] and hippocampal sclerosis [15]. Notably, the aberrant expression of SLC6A1 at both gene and protein levels have been also found in various human cancers. For example, SLC6A1 expression in mucosa of atrophic gastritis and duodenal metaplasia were up-regulated by more than 10 times as compared with normal gastric mucosa [16],; SLC6A1 overexpression was reported to promote cell growth and metastasis of clear cell renal cell carcinoma(CCRCC) [17] and was related to drug resistance to topotecan of ovarian cancer cell line [18].

To the best of our knowledgement, the expression patterns and the clinical significance of SLC6A1 in prostate cancer, as well as its associations with prostate cancer progression and patients’ response to chemotherapy remain unknown. To address these problems, we here examined the expression patterns of SLC6A1 protein in PCa tissues by immunohistochemistry based on Tissue microarray. Associations of SLC6A1 protein expression with various clinicopathological features and patients’ prognosis of PCa were also statistically evaluated based on TCGA data. Roles of SLC6A1 deregulation in prostate carcinogenesis and drug resistance was further determined in vitro and in vivo experiments.

## Methods

### Ethic statement

This study was approved by the human study ethics committees at Guangzhou First People’s Hospital and Guangzhou Medical University of P. R. China. All specimens were handled and made anonymous according to the ethical and legal standards.

The mice were purchased from experimental animal center of Sun Yat sen University, and were killed by cervical dislocation. All animal experiments in this study were performed in compliance with the guidelines of the Institute for Laboratory Animal Research at Guangzhou Medical University, Guangzhou, P. R. China.

### Patients and tissue samples

Tissue microarrays (TMA) included 50 prostate cancer tissues and 30 non-cancerous prostate tissues obtained from 80 consecutive prostate cancer patients purchased from Xi’an Ailina Biotechnology Co, Ltd. (Xi’an, People’s Republic of China; catalog number: PR807c). Patients who received adjuvant or neoadjuvant hormonal or radiation treatment prior to cancer recurrence were excluded. All hematoxyolin-eosin (H&E)-stained sections from each case were reviewed, and the Gleason score was reassigned according to the current grading recommendation provided by the International Society of Urological Pathology. Immunoreactivity scores (IRS) is the sum of the percentage and the intensity score. Staining was scored in accordance with the percentage and intensity of staining. The scores of staining percentage were as follows: 0 (≤5%), 1 (5–30%), 2 (30–70%), 3 (≥70%); the scores of staining intensity were as follows: 0 (negative), 1 (weak), 2 (medium) and 3 (strong). The final IRS (0–6) is the sum of percentage score plus intensity score. The median value of all SLC6A IRSs was used as a cutoff in this study. In addition, we also collected the TCGA dataset and the Taylor dataset, which both are publicly available datasets including 498 and 113 primary prostate cancer patients with the expression data of *SLC6A1* mRNA, respectively. Table 1 summarized the clinicopathological data of prostate cancer patients enrolled in this study and the TCGA dataset, including age, Gleason score, clinical stage, metastasis status and PSA failure status.

**Table 1:** Associations of SLC6A1 protein expression with various clinicopathological characteristics of PCa patients

Clinicopathological Characteristics	IRS of SLC6A1 in our cohort (n, %)				SLC6A1 expression in TCGA dataset		
	Case	Low (*n* = 11)	High (*n* = 39)	*P*	Case	Mean ± S.D.	*P*
Age(years)							
< 66	20	7(35.0)	13(65.0)	0.070	355	15.05 ± 13.76	0.995
≥ 66	30	4(13.3)	26(86.7)		143	15.04 ± 10.67	
Gleason score							
<8	31	6(19.4)	25(80.6)	0.564	292	12.47 ± 10.40	< 0.001
≥ 8	19	5(26.3)	14(73.7)		206	18.69 ± 15.15	
Clinical stage							
<T3A	29	5(17.2)	24(82.8)	0.340	194	12.22 ± 10.25	< 0.001
≥ T3A	21	6(28.6)	15(71.4)		304	16.85 ± 14.12	
Metastasis							
No		–	–		416	14.45 ± 12.07	0.059
Yes		–	–		82	18.09 ± 16.42	
PSA failure							
No		–	–		439	14.57 ± 12.69	0.042
Yes		–	–		59	18.62 ± 14.31	

Note: “–” No cases in our cohort

### Cell culture and transfection

Two human prostate cancer cell lines, LNCaP and PC3, were purchased from the American Type 6 Culture Collection (Manassas, VA, USA) and were cultured in DMEM medium (Cat No.:SH30022.01B, Hyclone, USA) supplemented with 10% fetal bovine serum (Cat No.:10270–106, Gibico, USA), 100 U/mL penicillin and 100 μg/mL of streptomycin. All cell lines were maintained at 37 °C in a humidified chamber supplemented with 5% CO_2_.

To enforce the expression of SLC6A1 in prostate cancer cells, the SLC6A1 coding sequence cloned into pLV.O(Provided by Huiyuanyuan company of China)/ blank vector (NC) were transfected into prostate cancer cells using Lipofectamine 2000 Reagent (Cat No: 11668019, Invitrogen, USA) according to the manufacturer’s protocol. The shRNA-SLC6A1-c was constructed with the target sequence as GCTGTTGATGCTGGGCATT, and the pre-SLC6A1 was cloned in the ORF sequence of SLC6A1(NM_003042.4). Forty-eight hours after the transfection, prostate cancer cells were collected and used for the functional analyses.

### Cell proliferation, cell cycle, invasion and migration assay

Functions of SLC6A1 in cell proliferation, cell cycle, invasion and migration of prostate cancer cells were evaluated by CCK-8, flow cytometry, transwell and the scratch wound-healing motility assays, respectively, according to our previous descriptions [19–21].

### Generation of the in vivo xenograft model

PC3 cells transfected with PRE-SLC6A1/PRE-NC or SH-SLC6A1/SH-NC were trypsinized and suspended in phosphate-buffered saline (PBS). Then, the cells were subcutaneously injected into the flanks of each nude mouse (10 per group). PC3 cells were subcutaneously injected at a concentration of 4 × 10^6^cells reaching a total capacity of 100 μL. On day 42, the mice were sacrificed. Tumor volume was calculated using the formula (W^2^ × L)/2, where W represented width and L represented length. The mice were manipulated and housed according to protocols approved by the Institute for Laboratory Animal Research at Guangzhou Medical University.

### Western blot analysis

Expression level of SLC6A1 protein in PCa cell lines with or without transfection were detected by Western blot analysis according to the protocol of our previous studies [19–21]. The used antibodies detail are: anti- SLC6A1rabbit monoclonal antibody (Cat No. bs-6617R, Bioss Co Ltd.,China), anti-β-actin rabbit monoclonal antibody (Cat No. BM0627, Boster, China), anti-GAPDH rabbit monoclonal antibody (Cat No. 10494–1-AP, Proteintech, USA).

### Immunohistochemistry

Cellular distribution and expression level of SLC6A1 protein in clinical PCa tissues, as well as those of PCNA and MMP-9 proteins in subcutaneous tumor xenografts of nude mice were examined by immunohistochemistry according to our previous description [19–21]. The used antibodies detail are: anti-SLC6A1rabbit monoclonal antibody (CatNo.bs-6617R, Bioss Co Ltd.,China), anti-PCNA rabbit monoclonal antibody (CatNo.sc-56, Santa CruzCo Ltd., USA), anti-MMP9 rabbit monoclonal antibody (CatNo.sc-6840, Santa Cruz Co Ltd., USA), anti-β-actin rabbit monoclonal antibody (CatNo.BM0627, Boster, China), anti-GAPDH rabbit monoclonal antibody (CatNo.10494–1-AP, Proteintech, USA).

### Effects of docetaxel on prostate cancer cell

PC3 and LNCaP cells were respectively seeded onto 96-well plate and incubated with docetaxel at the concentration of 0.1 nM, 0.5 nM, 2.5 nM, 12.5 nM, 62.5nMfor 48 h. Then, the prostate cancer cell proliferation were evaluated by CCK-8 assay according to our previous descriptions [19–21]. Moreover, we used flow cytometry to detect the ROS of PCa cell lines which was labeled by DHE probe.

### Statistical analyses

All statistical analyses here were performed by SPSS software for Windows (version 22.0, SPSS Inc., IL, USA). Data of continuous variables were expressed as mean ± SD. Associations of SLC6A1 expression with various clinicopathological parameters were evaluated by Fisher’s exact test for any 2 × 2 tables and Pearson χ^2^ test for non-2 × 2 tables. Survival analysis was performed by Kaplan-Meier method and Cox regression model. For functional analyses in vitro and in vivo, the differences between groups were analyzed using a Student t test when comparing only two groups or one-way analysis of variance when comparing more than two groups. Differences were considered statistically significant when the *P* value was less than 0.05.

## Results

### SLC6A1 overexpression significantly associates with aggressive progression and poor prognosis in patients with prostate cancer

**Fig. 1:**
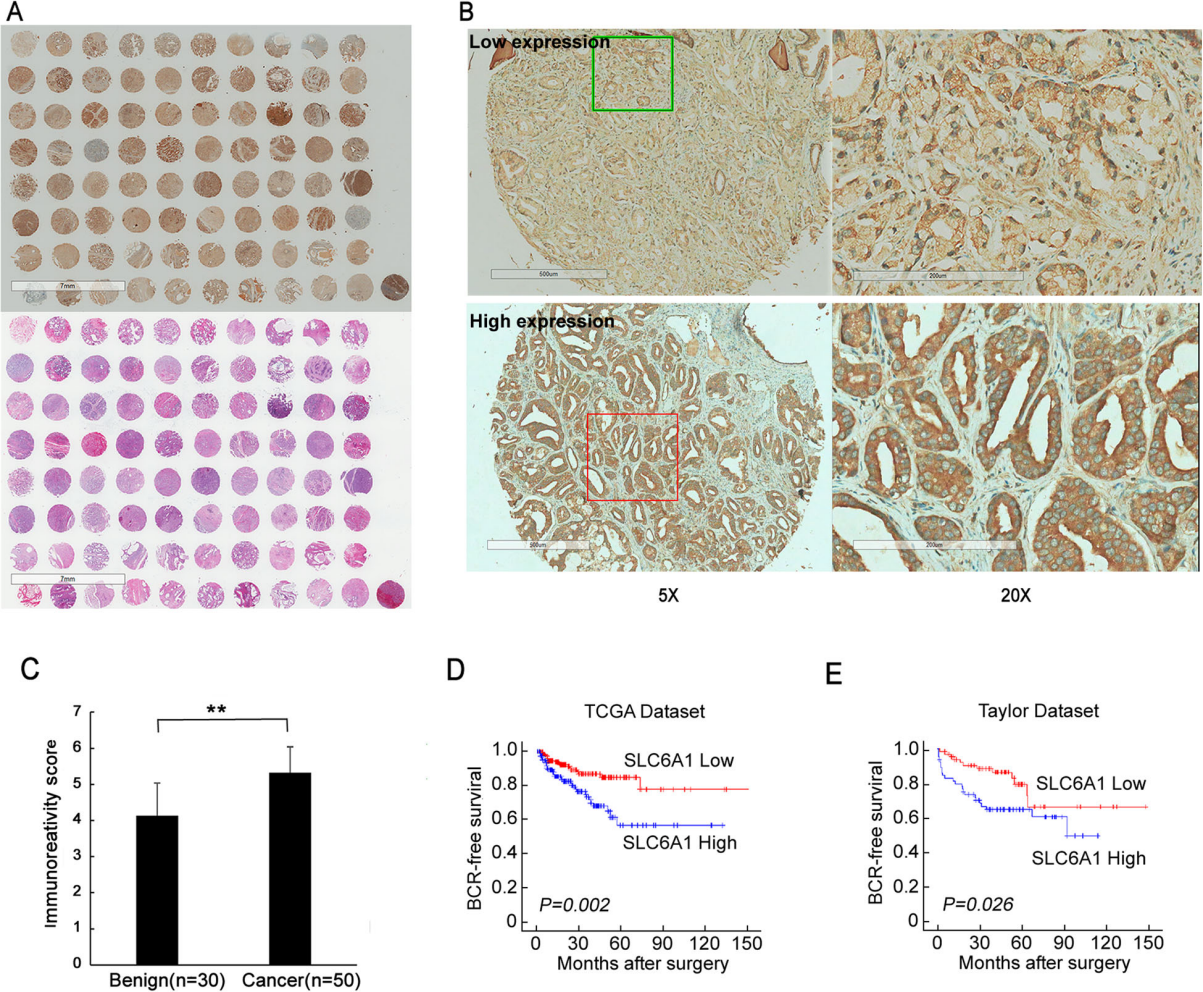
SLC6A1 overexpression significantly associates with aggressive progression and poor prognosis in patients with prostate cancer (**a**). A full view of the immunohistochemistry staining and HE staining for SLC6A1 in our TMA cohort. **b** Immunostaining of highSLC6A1 expression and low SLC6A1expression. Magnification of the right panel is × 5; the left panel is × 20. **c** Immunoreativity score (IRS) of SLC6A1 expression in the benign group and the cancer group. There was a obvious difference in SLC6A1 expression between two groups(*P* = 0.004). **d** There was a significant difference in BCR-free survival between patients with high and low SLC6A1 mRNA expression in TCGA database (*P* = 0.002). **e** There was also a significant difference in BCR-free survival between patients with high and low SLC6A1 mRNA expression in Taylor database (*P* = 0.026)

As shown in Fig. 1a-b, positive immunostaining of SLC6A1 protein was localized in the cell cytoplasm of prostate cancer tissues. The median value of SLC6A1 immunoreactive score (IRS) was used as a cutoff point to divided all samples into high or low SLC6A1 expression groups. Statistically, the frequency of high SLC6A1 protein expression in prostate cancer tissues was significantly higher than that in benign tissues (cancer vs. benign:78.0% vs.46.7%, *P* = 0.004, Fig. 1c). In addition, the associations of SLC6A1 expression with various clinicopathological characteristics were evaluated based on both our cohort and TCGA dataset. As shown in Table 1, the expression levels of SLC6A1 mRNA in prostate cancer patients with high Gleason score (*P* < 0.001), advanced clinical stage (*P* < 0.001) and positive PSA failure (*P* = 0.042) were significantly higher than those with low Gleason score, early clinical stage and negative PSA failure. However, there were no significant differences in our cohort, which might be due to the relative small clinical cohort used in this study.

In survival analysis, pairwise comparisons showed significant differences in the metastasis-free survival (for the TCGA dataset: *P* = 0.002, Fig. 1d; for the Taylor dataset: *P* = 0.026, Fig. 1e) between prostate cancer patients with high SLC6A1and low SLC6A1 expression. Further univariate analysis using a COX regression model revealed that SLC6A1 expression (*P* = 0.005, Table 2), Gleason score (*P* = 0.0001, Table 2), pathological tumor stage (*P* = 0.0001, Table 2) and clinical stage (*P* = 0.001, Table 2) were significantly associated with metastasis-free of prostate cancer patients. Among them, Gleason score (*P* = 0.005) and clinical stage (*P* = 0.021) were demonstrated to be independent prognostic factors for this cancer (Table 2) according to the multivariate analysis.

**Table 2:** Prognostic value of SLC6A1 expression for the biochemical recurrence-free in univariate and multivariate analyses using Cox Regression models

	Univariable Analysis	***P***	Multivariable Analysis	***P***
	HR(95%CI)		HR(95%CI)	
Age (< 66 vs. ≥6a6)	1.02 (0.575–1.820)	0.938	0.711 (0.373–1.356)	0.301
Gleason score (< 8 vs. ≥8)	3.927(2.182–7.070)	**0.0001**	2.619(1.344–5.106)	**0.005**
Pathological tumor stage (T2 vs. ≥T3)	4.800(2.060–11.185)	**0.0001**	2.092(0.826–5.299)	0.120
Clinical tumor stage (<T2A vs. ≥T2A)	3.289(1.647–6.568)	**0.001**	2.328(1.134–4.777)	**0.021**
SLC6A1 (high vs. low)	2.190(1.273–3.767)	**0.005**	1.751(0.977–3.138)	0.060

### SLC6A1 overexpression promotes proliferation, cell cycle, migration and invasion of prostate cancer cells

**Fig. 2:**
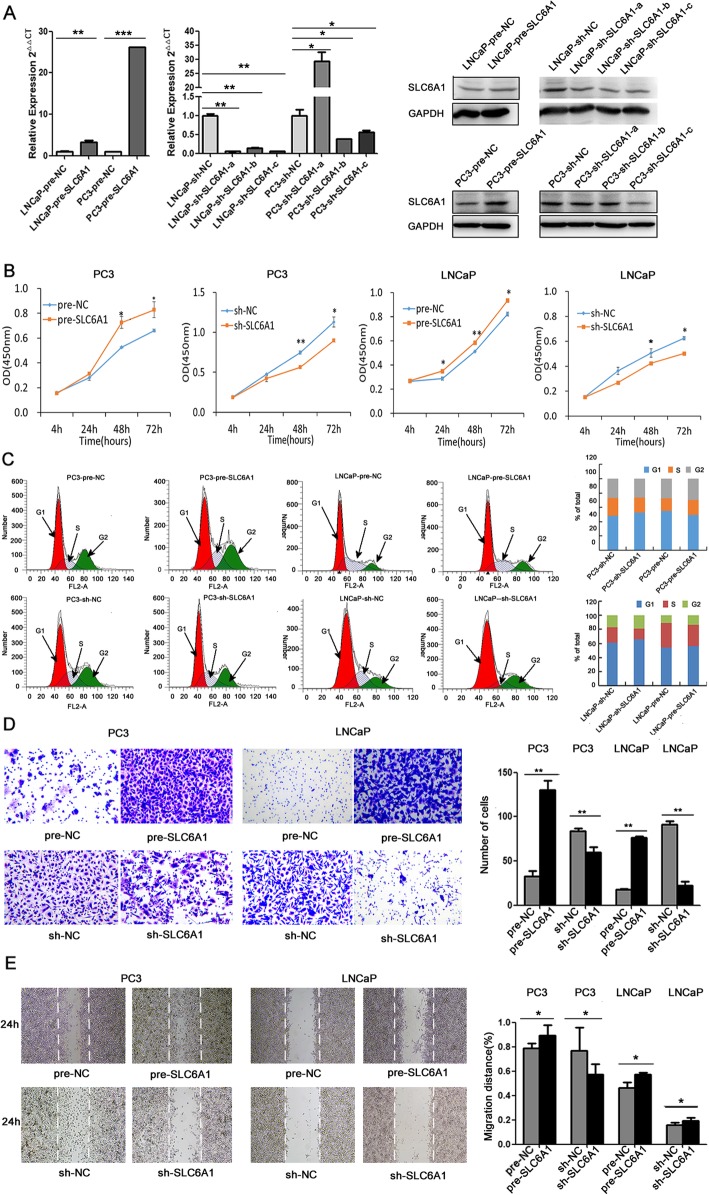
SLC6A1 overexpression promotes proliferation, migration and invasion of prostate cancer cell lines PC3 and LNCaP. **a** Both qPCR and Western blot analyses confirmed that the cell lines were successfully established. We choose the PC3-sh-SLC6A1-c and LNCaP-sh-SLC6A1-c to establish cell lines PC3 and LNCaP with the specific shRNA. Full-length blots/gels are presented in Supplementary Figure 5, 6, 7, 8. **b** Cell proliferation of PC3 and LNCaP cells transfected with SLC6A1 expression vector or the specific shRNA examined by CCK-8 assay.**c** Cell cycle distribution of PC3 and LNCaP cells transfected with SLC6A1 expression vector or the specific shRNA examined by flow cytometry analysis. The chart showed the number of cell cycle distribution in different stages of PC3 and LNCaP cell lines. Histogram showing the percentages of cells in each phase of the cell cycle which was analyzed by ModFit LT (Verity Software). **d** Invasion activity of PC3 and LNCaP cells transfected with SLC6A1 expression vector or the specific shRNA examined by Transwell assays. **e** Migration activity of PC3 and LNCaP cells transfected with SLC6A1 expression vector or the specific shRNA examined by Wound-healing assays

To assess the effects of SLC6A1 in aggressive features of prostate cancer cells, we established stable cell lines PC3 and LNCaP with the overexpressed orthe reduced expression of SLC6A1 following the transfection of the expression vector or the specific shRNA, respectively. Both qPCR and Western blot analyses confirmed that the cell lines were successfully established (Fig. 2a). CCK-8 assay showed that the overexpressed SLC6A1 distinctly promoted the cell proliferation of prostate cancer cell lines (for PC3 and LNCaP cells: both *P* < 0.05, Fig. 2b). In addition, flow cytometry analysis for cell cycle distribution of prostate cancer cell lines showed that the increased expression of SLC6A1 significantly promoted the cell cycle (for PC3 and LNCaP cells: both *P* < 0.05, Fig. 2c). Transwell assays clearly revealed that enforced expression of SLC6A1 significantly enhanced the invasive activities of both PC3 and LNCaP cells compared with those of control cells (both *P* < 0.05, Fig. 2d). Wound-healing assays demonstrated that SLC6A1overexpression markedly increased the migratory abilities of both PC3 and LNCaP cells (both *P* < 0.05, Fig. 2e). In contrast, the knockdown of SLC6A1 expression with the specific shRNA inPC3 and LNCaP cell lines could dramatically reduce the abilities of cell proliferation, cell cycle, invasion and motility (Fig. 2b~e).

### SLC6A 1 overexpression promotes prostate cancer growth in vivo

**Fig. 3:**
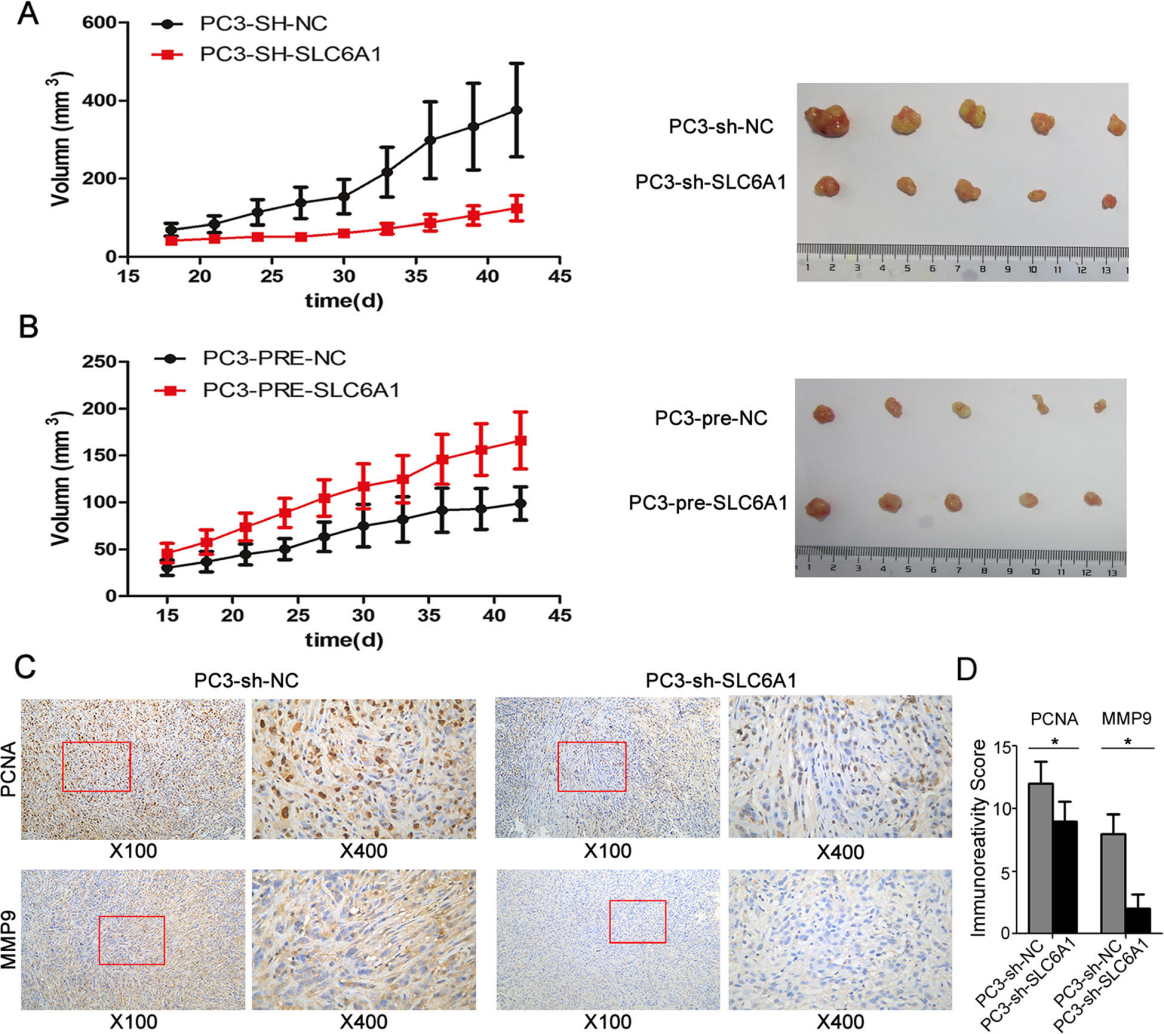
SLC6A1 overexpression promotes the tumor growth in vivo. **a** and **b** Comparison of tumor volumes obtained from different groups. Representative photographs of tumors in mouse models established using PC3 cells transfected with SLC6A1 expression vector or the specific shRNA. **c** Representative photographs of immunohistochemistry analysis on the expression levels of PCNA andMMP-9 proteins in tumor tissues obtained from mouse models established using PC3 cells transfected with SLC6A1 expression vector or the specific shRNA. Magnification of the left panel is × 100; the right panel is × 400. **d** The immunoreactivity score of the expression levels of PCNA andMMP-9 proteins in tumor tissues

To assess the effects of SLC6A1 in the carcinogenesis of the tumor xenografts, the in vivo xenograft model was established using PC3 cells transfected with SLC6A1 expression vector or the specific shRNA. As shown in Fig. 3a and b, the tumor volumes in SLC6A1 overexpression group were significantly larger than those in control group, while the reduced expression of SLC6A1 markedly suppressed the tumor growth of the mouse model (both *P* < 0.05). In addition, immunohistochemical analysis using cell growth marker PCNA and MMP-9 antibodies were performed. As shown in Fig. 3c and d, the immunostaining of PCNA and MMP-9 proteins in the tumor xenografts established by SLC6A1 expression vector-transfected PC3 or LNCaP cells were markedly stronger than that in the control groups (both *P* < 0.05). In contrast, the expression levels of PCNA and MMP-9 proteins in tumor tissues of the subcutaneous models bearing sh-SLC6A1-transfected PC3 cells were significantly decreased in the comparison with the control groups (both *P* < 0.05, Fig. 3c and d).

### SLC6A1 overexpression promotes the resistance to docetaxel of prostate cancer cells

**Fig. 4:**
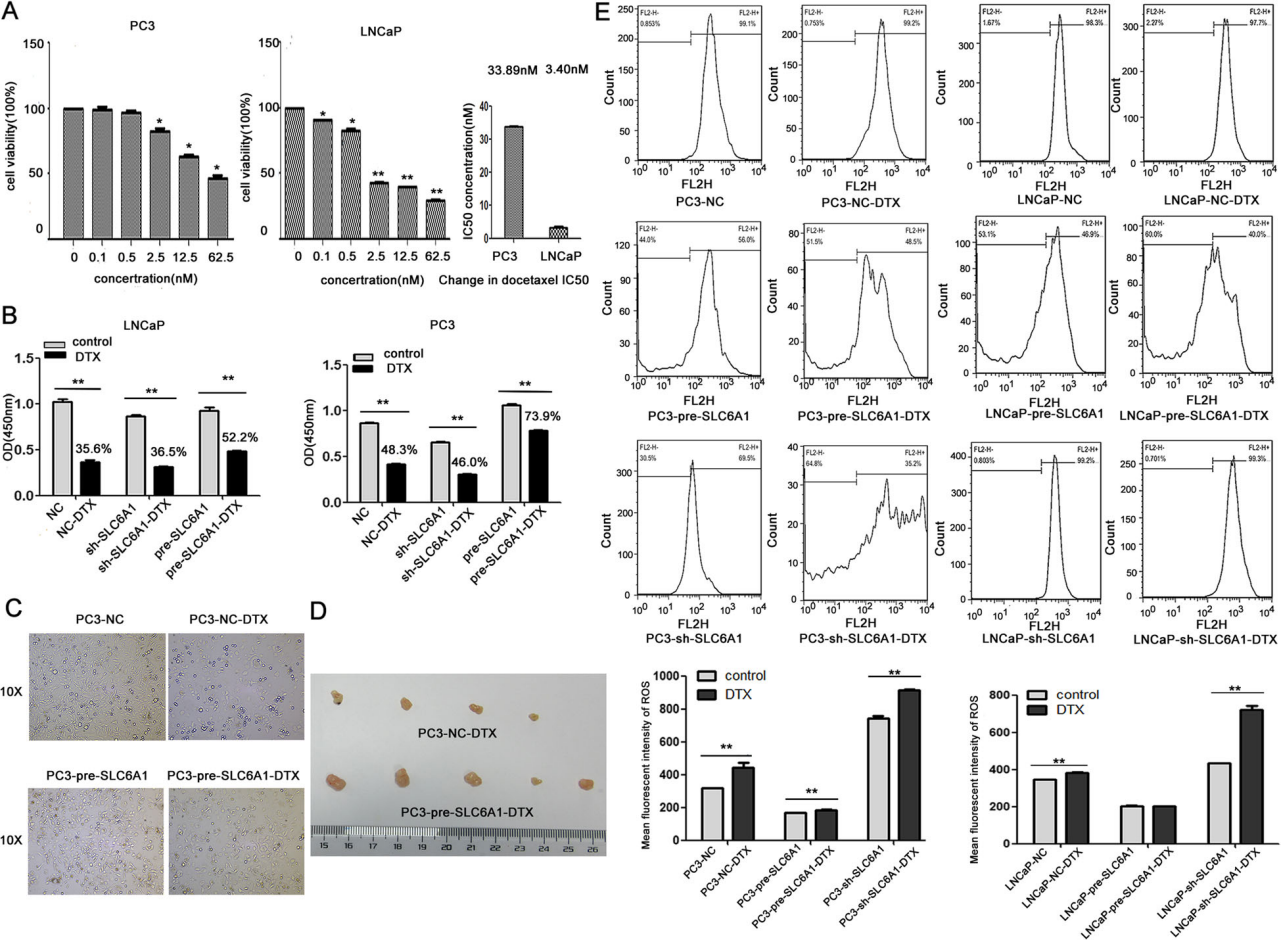
SLC6A1 overexpression promotes the resistance to docetaxel of prostate cancer cells. **a** Cell proliferation of PC3 and LNCaP cells treated with docetaxel in different concentrations. The IC50 concentration of PC3 cells was 33.89 nM, and the IC50 concentration of LNCaP cells was 3.40 nM. **b** Cell proliferation of PC3 and LNCaP cells transfected with SLC6A1 expression vector or the specific shRNA, and also treated with docetaxel in its IC50 concentration. **c** After treatment with docetaxel at IC50 concentration, the cell number and morphological changes of different PC3 cells were observed under microscope(X10). **d** Volume changes of PC3 transplanted tumor in SLC6A1 overexpression group and control groups after docetaxel treatment. **e** Effect of Docetaxel on ROS in PCa cell lines

As shown in Fig. 4a, the treatment of docetaxel significantly inhibited the cell proliferation of both PC3 and LNCaP cells with the IC50 concentrations of 33.89 nM and 3.40 nM, respectively. After treatment with docetaxel at IC50 concentration, the changes into cell proliferation of PC3 and LNCaP cells transfected with SLC6A1-expression-vectorwere significantly smaller than those transfected with SLC6A1-specific shRNA (both *P* < 0.05, Fig. 4b).Under the microscope(X10), we found that the percentage of the dead cells in the SLC6A1-overexpression group was markedly lower than that in the control group(Fig. 4c).Moreover, the treatment with docetaxel could not reduce the tumor volumes of xenografts established using PC3 cells transfected with SLC6A1 expression vector significantly when compared with the control groups(Fig. 4d). ROS decreased largely in the SLC6A1 overexpression of PC3 and LNCaP cell lines, and increased obviously in the SLC6A1 low-expression group whose ROS added more after the treatment of docetaxel (Fig. 4e).

## Discussion

Despite novel hormonal drugs, such as enzaluram and abiraterone, docetaxel still plays a crucial role in the treatment of CRPC [22, 23]. It can effectively reduce the level of PSA, gain survival benefits and prolong the overall survival of CRPC patients. However, there were more than half of CRPC patients still showing no response to docetaxel chemotherapy [8]. Therefore, it is necessary to screen out the patients with docetaxel chemotherapy resistance.

In this study, the immunohistochemical analysis based on TMA revealed a significantly increased expression of SLC6A1 protein in prostate cancer tissues compared with non-cancerous prostate tissues (*P* = 0.004). According to the TCGA and Taylor datasets, we demonstrated that the high expression of SLC6A1 mRNA was associated with advanced cancer progression (including high Gleason score and clinical stage, as well as positive PSA failure) and short biochemical recurrence-free survival. The significant clinical relevance of SLC6A1 in prostate cancer prompted us to investigate its molecular functions based on in vitro and in vivo experiments. Our data indicated that SLC6A1 may play an oncogenic role in prostate cancer cells via promoting cell proliferation, cell cycle, migration and invasion, which may be consistent with theprovious findings of Maolakuerban N [17]. Moreover, the enforced expression of SLC6A1 may promote the growth of the tumor xenografts, and also enhance the immunostaining of cell growth markers PCNA and MMP-9. To the best of our knowledge, this is the first investigation on the clinical significance and biological functions of SLC6A1 in prostate cancer.

As a kind of membrane transporters, the SLC family have been indicated to play crucial roles in regulating chemo-sensitivity of cancer cells by affecting the transport and the target genes of chemotherapy drugs through inherent gene changes [24–26] . For example,SLC9A2 and SLC16A6 was found to regulate the resistance to MTX [18]; SLC7A5 expression was associated with the resistance to melphalan [27]; SLC7A11 played a role in the resistance to cisplatin [28]. Notably, Januchowski R et al. [18] reported that SLC6A1 significantly increased resistance to topotecan chemotherapy of W1 ovarian cancer. Similar to this result, our data also demonstrated that the overexpression of SLC6A1 in prostate cancer may enhance in the resistance to docetaxel of prostate cancer.

SLC6A1, as a channel protein of chloride, can modulate the tansport of chloride, but has not direct downstream target genes, or there may be a second messenger that we have not discovered. As a group of important signal molecules, ROS(reactive oxygen species) plays a definite role in the development of cancer by affecting DNA damage, leading to the activation of oncogenes, or the loss of function of tumor suppressor gene [29]. In tumor cells, high concentration of ROS causes apoptosis and necrosis, while low concentration promotes tumor cell proliferation. The results showed that ROS decreased in overexpression group and promoted the proliferation of prostate cancer cells, while ROS increased in inhibition group, which could lead to apoptosis and necrosis of tumor cells. It was confirmed that overexpression of SLC6A1 can promote the proliferation of prostate cancer cells. Sometimes, ROS is closely related to the sensitivity or drug resistance of anticancer drugs [30]. Taxanes induced reactive oxygen species production in cancer cells which may influence the cell death or the drug resistance [31]. Our experiments have found that docetaxel can increase ROS in PC3 and LNCaP cells in vitro. However, we found that ROS decreased obviously when docetaxel acted on PC3 and LNCaP cells overexpressing SLC6A1, resulting in drug resistance. Therefore, the increase of ROS may be a factor leading to drug resistance.

## Conclusion

In conclusion, our data suggest that SLC6A1 overexpression may be associated with aggressive progression and short biochemical recurrence-free survival of prostate cancer, and may be related to the resistance to docetaxel therapy. Further research should be focus on the molecular mechanisms underlying the involvement of SLC6A1in prostate cancer.

## Supplementary information

**Additional file 1.**

**Additional file 2.**

**Additional file 3.**

**Additional file 4.**

**Additional file 5.**

**Additional file 6.**

## Data Availability

Not applicable.

## Abbreviations

IRS: Immunoreactivity scores
PCa: Prostate cancer
PSA: Prostate-specific antigen
SLC6A1: Solute Carrier Family 6 Member 1
CI: Confidence interval
HR: Hazard ratio

## References

[1] Ferlay J, Colombet M, Soerjomataram I (2018). Cancer incidence and mortality patterns in Europe: estimates for 40 countries and 25 major cancers in 2018. Eur J Cancer.

[2] Siegel RL, Miller KD, Jemal A (2015). Cancer statistics, 2015. CA Cancer J Clin.

[3] Saad F, Pantel K (2012). The current role of circulating tumor cells in the diagnosis and management of bone metastases in advanced prostate cancer. Future Oncol.

[4] Luo Q, Yu XQ, Smith DP, O'Connell DL (2015). A population-based study of progression to metastatic prostate cancer in Australia. Cancer Epidemiol.

[5] Hirst CJ, Cabrera C, Kirby M (2012). Epidemiology of castration resistant prostate cancer: a longitudinal analysis using a UK primary care database. Cancer Epidemiol.

[6] de la Taille A, Martínez-Piñeiro L, Cabri P, Houchard A, Schalken J (2017). Factors predicting progression to castrate-resistant prostate cancer in patients with advanced prostate cancer receiving long-term androgen-deprivation therapy. BJU Int.

[7] Wu KJ, Pei XQ, Tian G (2018). PSA time to nadir as a prognostic factor of first-line docetaxel treatment in castration-resistant prostate cancer: evidence from patients in northwestern China. Asian J Androl.

[8] Tannock IF, de Wit R, Berry WR (2004). Docetaxel plus prednisone or mitoxantrone plus prednisone for advanced prostate cancer. N Engl J Med.

[9] Abdul M, Hoosein N (2006). Reduced Kv1.3 potassium channel expression in human prostate cancer. J Membr Biol.

[10] Fixemer T, Wissenbach U, Flockerzi V, Bonkhoff H (2003). Expression of the Ca2+−selective cation channel TRPV6 in human prostate cancer: a novel prognostic marker for tumor progression. Oncogene.

[11] Gonzalez-Burgos G (2010). GABA transporter GAT1: a crucial determinant of GABAB receptor activation in cortical circuits. Adv Pharmacol.

[12] Yuan FF, Gu X, Huang X, Zhong Y, Wu J (2017). SLC6A1 gene involvement in susceptibility to attention-deficit/hyperactivity disorder: a case-control study and gene-environment interaction. Prog Neuro-Psychopharmacol Biol Psychiatry.

[13] Palmer S, Towne MC, Pearl PL (2016). SLC6A1 mutation and Ketogenic diet in epilepsy with myoclonic-atonic seizures. Pediatr Neurol.

[14] Hoftman GD, Volk DW, Bazmi HH, Li S, Sampson AR, Lewis DA (2015). Altered cortical expression of GABA-related genes in schizophrenia: illness progression vs developmental disturbance. Schizophr Bull.

[15] Schijns O, Karaca Ü, Andrade P (2015). Hippocampal GABA transporter distribution in patients with temporal lobe epilepsy and hippocampal sclerosis. J Chem Neuroanat.

[16] Kim KR, Oh SY, Park UC (2007). Gene expression profiling using oligonucleotide microarray in atrophic gastritis and intestinal metaplasia. Korean J Gastroenterol.

[17] Maolakuerban N, Azhati B, Tusong H, Abula A, Yasheng A, Xireyazidan A (2018). MiR-200c-3p inhibits cell migration and invasion of clear cell renal cell carcinoma via regulating SLC6A1. Cancer Biol Ther.

[18] Januchowski R, Zawierucha P, Andrzejewska M, Ruciński M, Zabel M (2013). Microarray-based detection and expression analysis of ABC and SLC transporters in drug-resistant ovarian cancer cell lines. Biomed Pharmacother.

[19] Cai C, Chen QB, Han ZD (2015). miR-195 inhibits tumor progression by targeting RPS6KB1 in human prostate Cancer. Clin Cancer Res.

[20] Lin ZY, Huang YQ, Zhang YQ (2014). MicroRNA-224 inhibits progression of human prostate cancer by downregulating TRIB1. Int J Cancer.

[21] Lin ZY, Chen G, Zhang YQ (2017). MicroRNA-30d promotes angiogenesis and tumor growth via MYPT1/c-JUN/VEGFA pathway and predicts aggressive outcome in prostate cancer. Mol Cancer.

[22] Thomas C, Brandt MP, Baldauf S (2018). Docetaxel-rechallenge in castration-resistant prostate cancer: defining clinical factors for successful treatment response and improvement in overall survival. Int Urol Nephrol.

[23] Teply BA, Hauke RJ (2016). Chemotherapy options in castration-resistant prostate cancer. Indian J Urol.

[24] Nakanishi T (2007). Drug transporters as targets for cancer chemotherapy. Cancer Genomics Proteomics.

[25] Huang Y (2007). Pharmacogenetics/genomics of membrane transporters in cancer chemotherapy. Cancer Metastasis Rev.

[26] Huang Y, Sadée W (2006). Membrane transporters and channels in chemoresistance and -sensitivity of tumor cells. Cancer Lett.

[27] Uchino H, Kanai Y, Kim DK (2002). Transport of amino acid-related compounds mediated by L-type amino acid transporter 1 (LAT1): insights into the mechanisms of substrate recognition. Mol Pharmacol.

[28] Okuno S, Sato H, Kuriyama-Matsumura K (2003). Role of cystine transport in intracellular glutathione level and cisplatin resistance in human ovarian cancer cell lines. Br J Cancer.

[29] Hahn WC, Weinberg RA (2002). Rules for making human tumor cells. N Engl J Med.

[30] Trachootham D, Alexandre J, Huang P (2009). Targeting cancer cells by ROS-mediated mechanisms: a radical therapeutic approach. Nat Rev Drug Discov.

[31] Kosaka T, Hongo H, Miyazaki Y, Nishimoto K, Miyajima A, Oya M (2017). Reactive oxygen species induction by cabazitaxel through inhibiting Sestrin-3 in castration resistant prostate cancer. Oncotarget.

