# Supplementary figures and images for: Overexpression of SLC6A1 associates with drug resistance and poor prognosis in prostate cancer
Source: BMC Cancer. 2020 Apr 6;20:289. doi: 10.1186/s12885-020-06776-7 (PMC7137497; doi:10.1186/s12885-020-06776-7)

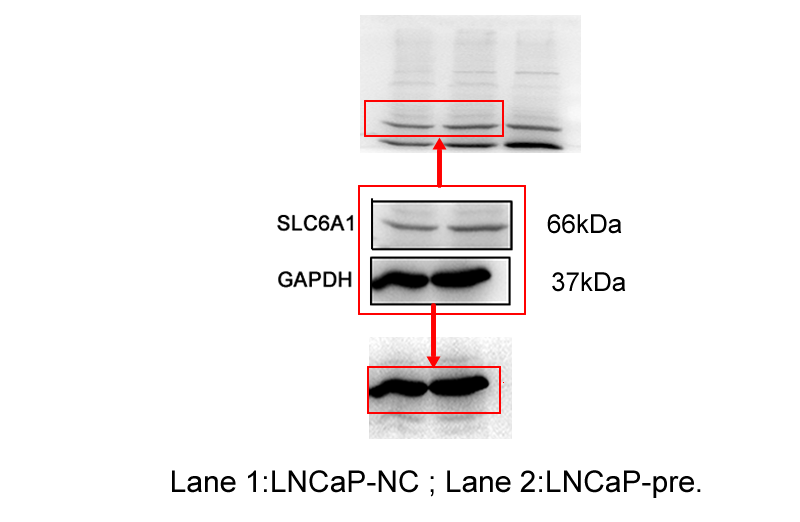

Supplement: Supplementary file 1 — Additional file 1. [file 12885_2020_6776_MOESM1_ESM.tif]

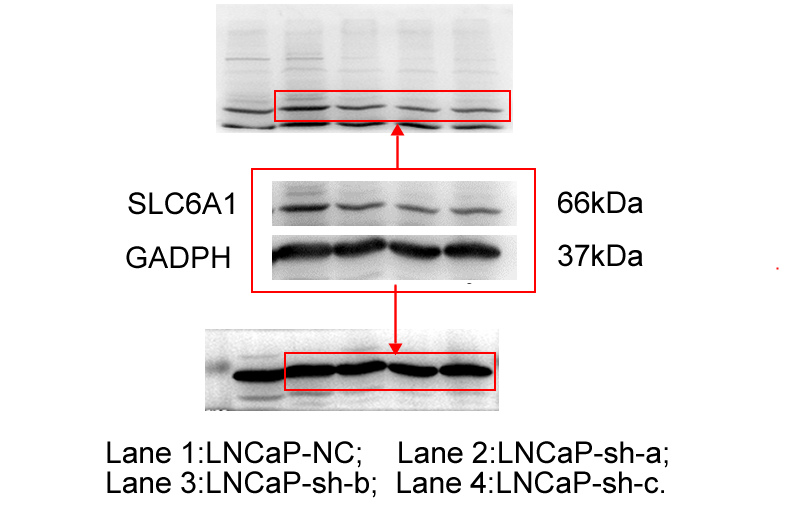

Supplement: Supplementary file 2 — Additional file 2. [file 12885_2020_6776_MOESM2_ESM.tif]

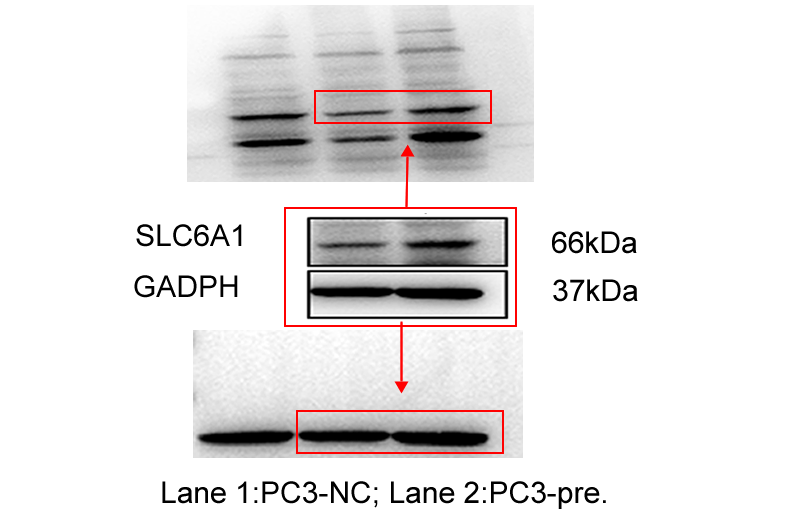

Supplement: Supplementary file 3 — Additional file 3. [file 12885_2020_6776_MOESM3_ESM.tif]

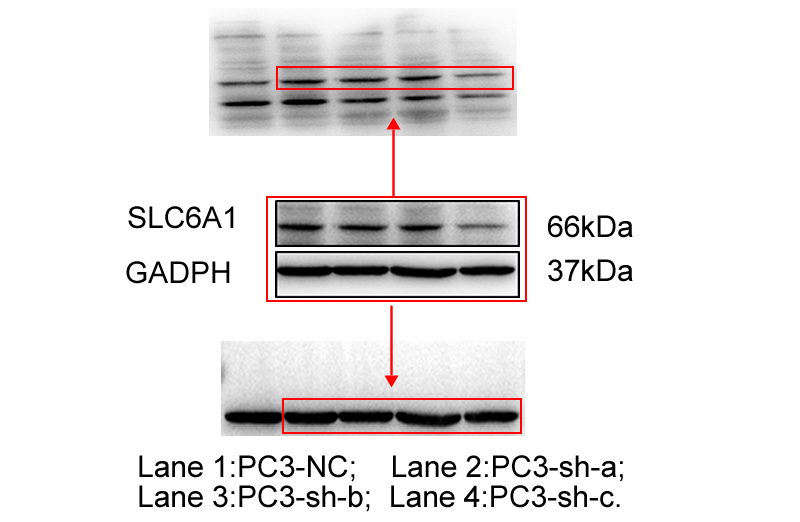

Supplement: Supplementary file 4 — Additional file 4. [file 12885_2020_6776_MOESM4_ESM.tif]
