# Supplementary material for: Overexpression of SLC6A1 associates with drug resistance and poor prognosis in prostate cancer
Source: BMC Cancer. 2020 Apr 6;20:289. doi: 10.1186/s12885-020-06776-7 (PMC7137497; doi:10.1186/s12885-020-06776-7)

Report of Human Cell Line Authentication

Delivery Date: Oct 21th, 2015

Analysis Date: Nov 3rd, 2015

Ⅰ. Sample

Sample Name: 'JD1124', labeled as 'LNCap', and was received on Oct 21th, 2015.

Ⅱ. Method and Procedure

1. PCR is amplified with STR Multi-amplification Kit (PowerPlex^{TM} 16 HS System);
2. PCR products are assayed with 3100 DNA Analyzer (Applied Biosystems®).

Ⅲ. Results

1. The results of the negative and positive control match expectations.
2. The STR profiles of the cell line sample are in the attached table and figure.

HCT116: ①Two loci (D5S818 and Penta_E) has tri-alleles. Contamination of other human cell line is not found. ②100% matched cell lines are found in ATCC and DSMZ data banks. And the cell line named as LNCap.FGC *et al*. ③The sample is a human cell line. Contamination of other species (*Cricetulus griseus, Macaca mulatta, Cercopithecus aethiops, Rattus norvegicus, Mus musculus, Bos Taurus*, IC) cells were not found in the sample.

Operator: Jiahao Jiang　　　　Auditor: Xuanyi Liang

Guangzhou Cellcook Biotech Co., Ltd

Table 1. STR profiles of LNCap cell line

	Allele1	Allele2	Allele3
D3S1358	15	16	
TH01	9		
D21S11	29	32. 2	
D18S51	11	12	
Penta_E	12	15	16
D5S818	10	11	12
D13S317	10	12	
D7S820	9. 1	10. 3	
D16S539	11		
CSF1PO	10	11	
Penta_D	12	12. 4	
AMEL	x	y	
vWA	16	18	
D8S1179	12	14	
TPOX	8	9	
FGA	19	20	

Figure 1. Authentication of the species of the sample

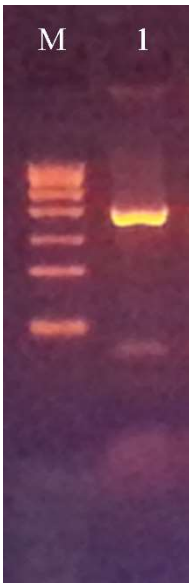

M: Marker. As the size of 700, 600, 500, 400, 300, 200 and 100bp from up to down.
1: The sample. The band size is 391bp which matches expectations.

Figure 2. STR profiles of LNCap cell line

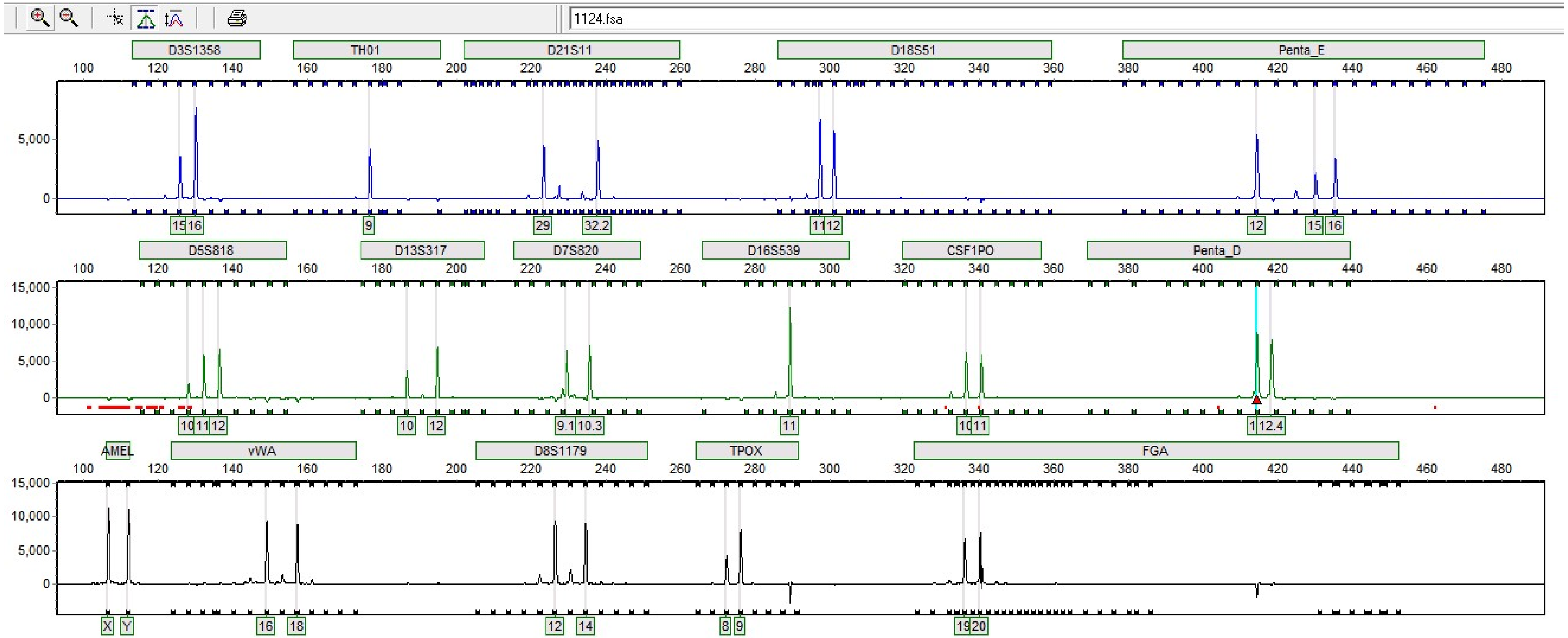

Supplement: Supplementary file 5 — Additional file 5. [file 12885_2020_6776_MOESM5_ESM.pdf]
