# Supplementary material for: Overexpression of SLC6A1 associates with drug resistance and poor prognosis in prostate cancer
Source: BMC Cancer. 2020 Apr 6;20:289. doi: 10.1186/s12885-020-06776-7 (PMC7137497; doi:10.1186/s12885-020-06776-7)
Supplement: Supplementary file 6 — Additional file 6. [file 12885_2020_6776_MOESM6_ESM.pdf]

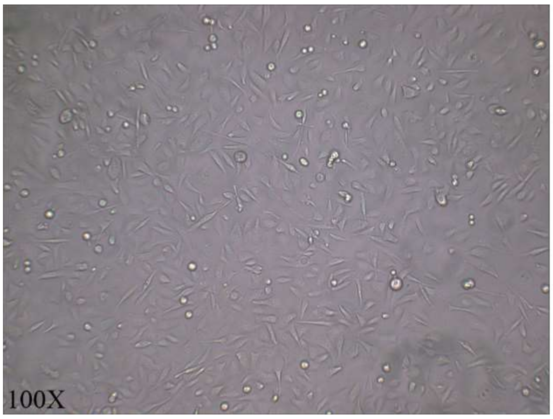

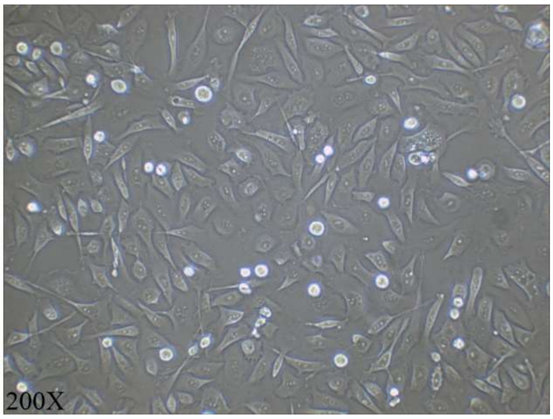

Catalog No.: CC1202

Cell Name: PC3

Size: T25 culture flask，1×10^6cells

Morphology: Epithelial

Culture Properties: Adherent

Characteristics: The PC-3 was initiated from a bone metastasis of a grade IV prostatic adenocarcinoma from a 62-year-old male Caucasian. The cells exhibit low acid phosphatase and testosterone-5-alpha reductase activities.

Culture Method: F-12K/RPMI-1640 10%FBS

Subcultivation Ratio: 1:3-1:6；Twice per week

Trypsined Time: 3-5 minutes

Lot: 20160717

STR Profile:

STR Profile	AMEL	CSF1PO	D13S317	D16S539	D5S818	D7S820	TH01	TPOX	vWA
PC3	X	11	11	11	13	8 11	6 7	8 9	17

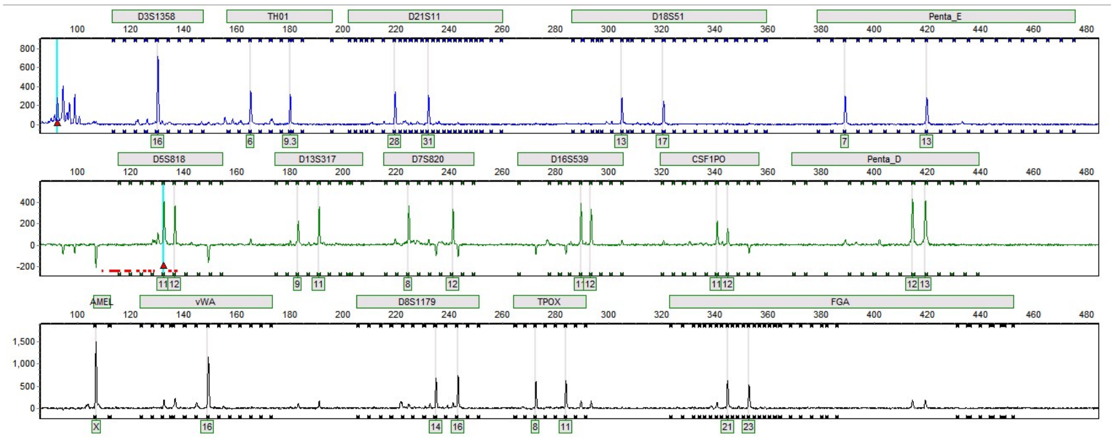

Address：R&D Unit 602, Stadard Property Unit 3, International Biotech Island, Guangzhou
Web: www.cellcook.com Tel：+8620-84298069 E-mail：info@cellcook.com
